# Supplementary material for: Personalized Cancer Medicine in the Media: Sensationalism or Realistic Reporting?
Source: J Pers Med. 2021 Jul 28;11(8):741. doi: 10.3390/jpm11080741 (PMC8399271; doi:10.3390/jpm11080741)
Supplement: Supplementary file 1 [file jpm-11-00741-s001.zip › jpm-1231979-supplementary.pdf]

Supplemental Table S1. Domains of coding instrument

Domain	Example(s)
Article Characteristics	Article source
Personalized Medicine	Definition, benefits, and challenges of personalized medicine
Genetic/Genomic Testing	Genetic testing delivery method (direct-to-consumer, clinic, research), specific genetic tests mentioned, whether GINA legislation was mentioned
Cancers and Other Diseases	Specific cancers mentioned
Targeted Therapies	Specific targeted therapies mentioned
Genetic Testing and Treatment Exemplars	Personalized stories of genetic testing

Supplemental Results:

Supplemental Figure S1. Genomic technologies reported (n = 287)

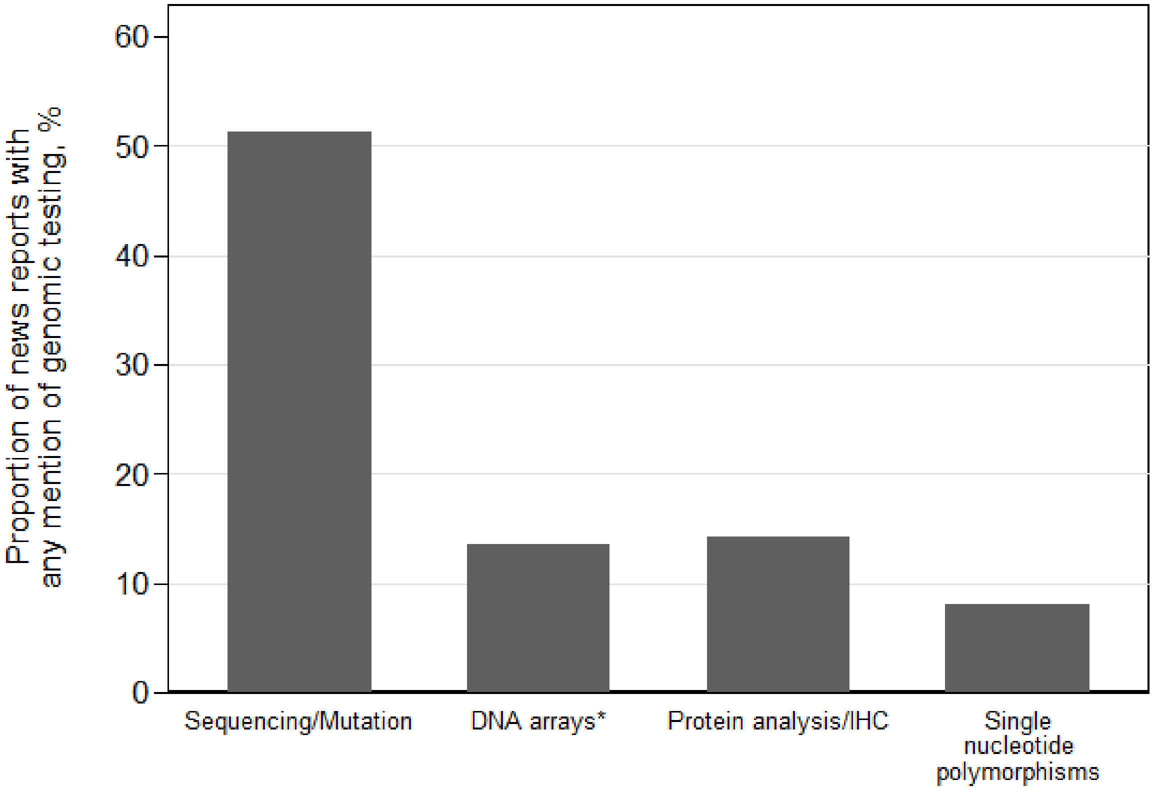

*Includes gene chips, microarrays, and gene expression profiling (ex. OncotypeDx)

Other Category of Genetic Tests: The "Other" category of genetic tests includes one or more references to the following: ACTN3; Amplichip; APOe; Calpain-10; CCR5; COMT; DRD4/2; ES01; fragile X; Genelex; HapK; HLA-B*5101; HLA-DR4; JAK3; KIF6; KIT; MART-1; MEF2A; MTHFR; P53; rs2180439; SERT; TCF7L2; TMPT; TPMP; TPMT; VKORC1; XIAP

Other Category of Targeted Therapies: The "Other" category of targeted therapies includes one or more references to the following: Abacavir; Alimta; Anthracycline; anti-clot; anti-depressants; Apo2L/Trail; Aurimune; Azathioprine; BiDil; Bucindolol; Campostar; Celexa; Centredekin; Clopidogrel; corticosteroids; COX-2; Dasatinib; Irinotecan; Lipitor; Maraviror; Nexavar; Omnitarg; Oncophage; Osteoprotegerin; PABP Inhibitors; PF299804; Plavix; PLX4032; Primaquine; Prozac; Rituxan; Sprycel; ST1-571; Tykerb; Velcade; Xeloda; Zevalin
For supplement:

Other Category of Cancer: "Other" category of types of cancer includes one or more references to the following: Adrenal cancer, bile-duct cancer, bronchoalveolar cancer, epithelial cancer, glaucoma, liver cancer, neuroblastoma, and stomach cancer.

Other Category of Diseases: The "Other" category of other diseases includes one or more references to the following: Abdominal aortic aneurysm, ADHD, aging, allergies, argininosuccinic acidemia, atherosclerosis, autoimmune, birth defects, blinding diseases, brain injury, bronchitis, burns, carpal tunnel, cataracts, chronic non-infectious diseases, congenital anomalies, Crohn's disease, dental, dermatological acne, Down Syndrome, dyslexia, enzyme deficiencies, eye disease, Fibiodysplasia Ossificans Progressiva, Gaucher disease, Fabry disease, genetic immune system mutation, Graves' disease, gum disease, hearing loss, Huntington's disease, immune, infertility, inflammation, insomnia, kidney failure, learning disabilities, liver, lower back problems, lupus, macular degeneration, memory loss, metabolic disorders, near-sightedness, neuromuscular disease, organ transplantation, 'other diseases', psoriasis, restless leg syndrome, retinal occlusion, sepsis, SIDS, skin ulcers, spina bifida, spinal muscular atrophy, Tay-Sachs, wounds
